# An Enhanced Drought-Tolerant Method Using SA-Loaded PAMPS Polymer Materials Applied on Tobacco Pelleted Seeds

**DOI:** 10.1155/2014/752658

**Published:** 2014-08-27

**Authors:** Yajing Guan, Huawei Cui, Wenguang Ma, Yunye Zheng, Yixin Tian, Jin Hu

**Affiliations:** ^1^Seed Science Center, College of Agriculture and Biotechnology, Zhejiang University, Hangzhou 310058, China; ^2^Yuxi Zhongyan Tobacco Seed Co., Ltd., Yuxi 653100, China

## Abstract

Drought is one of the most important stress factors limiting the seed industry and crop production. Present study was undertaken to create novel drought-resistant pelleted seeds using the combined materials with superabsorbent polymer, poly(2-acrylamide-2-methyl propane sulfonic acid) (PAMPS) hydrogel, and drought resistance agent, salicylic acid (SA). The optimized PAMPS hydrogel was obtained as the molar ratio of 2-acrylamido-2-methyl-propanesulfonic acid (AMPS) to potassium peroxydisulfate (KPS) and N, N′-methylene-bis-acrylamide (MBA) was 1 : 0.00046 : 0.00134. The hydrogel weight after swelling in deionized water for 24 h reached 4306 times its own dry weight. The water retention ratio (RR) of PAMPS was significantly higher as compared with the control. It could keep as high as 85.3% of original weight after 30 min at 110°C; even at 25°C for 40 d, the PAMPS still kept RR at 33.67%. PAMPS disintegration ratio increased gradually and reached around 30% after embedding in soil or activated sludge for 60 d. In addition, there were better seed germination performance and seedling growth in the pelleted treatments with SA-loaded PAMPS hydrogel under drought stress than control. It suggested that SA-loaded PAMPS hydrogel, a nontoxic superabsorbent polymer, could be used as an effective drought resistance material applied to tobacco pelleted seeds.

## 1. Introduction

Tobacco (*Nicotiana tabacum* L.) originated from tropical and subtropical area with abundant rainfall and high humidity and its water requirement is very high. Most of the tobacco planting regions often lack necessary irrigation facilities; hence, the water deficiency has become the major stress factor with high potential impact on tobacco yield. Seed pelleting technique is a new method of seed treatment. Presently, the planting ratio of tobacco pelleted seeds in china has reached around 90% [1]. Therefore, research and development on tobacco pelleted seed with drought resistance are of great significance.

So far, a few researches on drought-resistant coating seeds put forward to add some water absorbent into the coating agent [2]. When field irrigation or rainfall comes, the water absorbent could absorb water from the soil to form a small “reservoir,” and the stored water will come out of the small “reservoir” to be utilized by the seeds or seedlings when soil is too dry, to achieve drought resistance. However, the water absorption ratios of traditional water absorbents, such as polyacrylate and acrylic copolymer, were usually in the range from tens to hundreds [3–6]; the limited water absorption capacity could not give full play to the role of the small “reservoir.” In addition, it is just a single physical method to improve seed drought resistance, and the water absorbent is difficult to play the role of “reservoir” when there is lack of rainfall or irrigation; the application of coating seeds in arid, semi-arid, or other areas of less rainfall is limited. Therefore, combining the water absorbent and drought-resistant agents to form novel drought-resistant materials is particularly important to ensure the maximum seed germination and seedling establishment under drought stress.

Superabsorbent polymers (SAP) are new functional polymer materials containing strong hydrophilic groups that can absorb and retain extremely large amounts of a liquid relative to their own mass [7]. Based on the characteristics of high water absorption rate and good water retention, they are widely applied in agriculture, forestry, gardening, petroleum chemical industry, medical health, environment governance, and other fields [8, 9].

2-acrylamide-2-methyl propane sulfonic acid (AMPS) is a kind of multifunctional water-soluble anionic monomer. Due to the molecular structure of unsaturated double bond vinyl and the sulfonic acid functional group with strong anion and hydrophily, AMPS has good performance of polymerization [10] and the water absorption ratio of its polymers; it is known as a superabsorbent resin and can amount the thousands times of its own dry weight in general. The AMPS monomer has been widely applied in the synthesis of water treatment agents, adsorption and separation materials, and water absorbing and retention materials in high efficiency [10]. However, at present, the information on the application of the AMPS monomer on pelleting seeds is lacking in literature.

Salicylic acid (SA) is widely regarded as an endogenous plant growth regulator which plays a significant role in the signal transduction pathway of abiotic stresses in plants [11]. Exogenous SA-induced enhancement in the resistance to drought have been observed in many plants such as maize [12], wheat [13], and rice [14]. In a study, soybean seeds soaking in salicylic acid caused a positive effect on the accumulation of some ions and antagonists, or modifying the inhibitory effect of drought stress [15]. Sharafizad et al. [16] found that wheat seed soaking treatment with SA in low concentration at low level of drought stress could decrease the germination time and increase the germination percentage. However, the effect of SA loaded in superabsorbent polymers on plant drought resistance has not been reported.

The poly(2-acrylamide-2-methyl propane sulfonic acid) (PAMPS) hydrogel was prepared and its synthetic formula was optimized in this study. Owing to high water absorbability and unique three-dimensional network structure, PAMPS hydrogel is considered as a controlled release material by controlling the release of SA from coating agent and further improves the seedling drought tolerance. Therefore, PAMPS hydrogel was used in this study to determine the performance of superabsorbent resin, and SA was used as a drought-resistant chemical that was embedded into PAMPS resin. Then the enhanced drought-resistant effect of the SA-loaded PAMPS hydrogel as coating agent was studied.

## 2. Materials and Methods

### 2.1. Materials

Tobacco seeds of “Honghua Dajinyuan” (HHDJY) and “MSk326,” coating agent (talc and bentonite), and the adhesive were provided by Yunnan Provincial Academy of Tobacco Agricultural Sciences, China. 2-Acrylamido-2-methylpropane sulfonic acid (AMPS-H^+^) and salicylic acid (SA) were purchased from Shanghai Wing Science and Technology Co., Ltd., Shanghai, China. Potassium persulfate (KPS), N, N′-methylene-bis(acrylamide) (MBA), NaCl, and NaOH were obtained from Shanghai Dingguo Biotechnology Co., Ltd., Shanghai, China. All chemicals were used as received and experiments were carried out applying double distilled water (ddH_2_O).

### 2.2. The Synthesis of PAMPS

#### 2.2.1. The Preparation of Stock Solution

AMPS-H^+^ was crystallized from boiling methanol [17]. 2-Acrylamido-2-methylpropane sulfonic acid sodium salt (AMPS-Na^+^) stock solution was prepared by dissolving 35 g of AMPS-H^+^ in 70 mL of ddH_2_O followed by addition of 15 mL 30% w/v NaOH solution under cooling conditions. Afterwards, the solution was neutralized by titration with 1 M NaOH to pH 7.00. Finally, the volume of the solution was adjusted to 100 mL with ddH_2_O.

MBA was crystallized from warm acetone. The MBA stock solution was prepared by dissolving 10.5 g of MBA in about 90 mL of ddH_2_O and then adjusting the final volume to 100 mL with ddH_2_O (the concentration is 0.105 g/mL).

KPS stock solution was prepared by dissolving 0.070 g of KPS in 10 mL of ddH_2_O (the concentration is 0.007 g/mL).

For the hydrogel synthesis, ddH_2_O was cooled under nitrogen bubbling.

#### 2.2.2. Synthesis of Hydrogel

The copolymerization of PAMPS hydrogel was carried out in ddH_2_O at 40°C in the presence of KPS as the initiator (Table 1). AMPS, MBA, and KPS stock solutions were mixed together according to the ratios in Table 1. After bubbling nitrogen for 20 min, the glass tubes were sealed and immersed in 55°C water bath and the polymerization was conducted. The hydrogel would form well after 6~7 h.

### 2.3. The Characteristics of the Hydrogel

#### 2.3.1. Fourier Transform Infrared Spectroscopy Analysis

Fourier transform infrared spectroscopy (FTIR) was used to characterize the presence of specific chemical groups in the materials. 0.01 g of PAMPS powder was ground along with KBr to form a disc and analyzed by using FTIR absorbance mode. FTIR spectra were obtained in the range of wave number from 4000 to 500 cm^−1^ (Nicolet 5700, Thermo Nicolet, USA). The FTIR spectra were normalized and major vibration bands were associated with chemical groups.

#### 2.3.2. Swelling Measurements in Water and Salt Solutions

0.03 g (*m*_0_) of PAMPS powders was accurately weighed and added in air-tight test bottles containing 300 mL of ddH_2_O or 0.9% (w/w) NaCl solution. After every 30 min of PAMPS absorbing solution (totally 24 h) at room temperature (25°C), the residues of ddH_2_O or NaCl solution in the container were filtered with 250 micron nylon mesh filter bag and the PAMPS weight in the swollen state (*m*_*d*_) was measured. Its weight swelling ratio (SR) was calculated as SR = ((*m*_*d*_ − *m*_0_)/*m*_0_) × 100% (g/g), where the *m*_0_ and *m*_*d*_ are the weights of the hydrogels in dry state and swollen state for a given time, respectively.

#### 2.3.3. Water Retention Determination

Certain amount (*m*_0_, g) of swollen PAMPS (F8) (24 h) was measured in a beaker, and the same weight of water in another beaker was used as a control. Beakers were placed in a chamber at 25°C and their weights were measured (*m*_*d*_) every 5 days (totally 40 d) or they were kept in the chambers with different high temperatures (30, 50, 70, 90 and 110°C) for 30 min and then were weighted (*m*_*d*_). The water retention ratio (RR) at room temperature (25°C) or high temperatures was calculated as RR = (*m*_*d*_/*m*_0_) × 100% (g/g), where the *m*_0_ and *m*_*d*_ are initial swollen hydrogel weight and weights obtained in a given time at certain temperature, respectively.

#### 2.3.4. PAMPS Disintegration Ratio

Several pots were filled with disinfected soil containing 40% (w/w) water. A certain amount (*m*_0_, g) of dried PAMPS (F8) in the form of bar was embedded in soil and activated sludge. Then the pots were placed in a chamber at 25°C for 60 d, and the soil moisture level was maintained at 40% during the whole period. The PAMPS samples were collected every 10 d, rinsed gently with the ddH_2_O to remove impurities on its surface, and finally dried at 60°C to its constant weight (*m*_*d*_). The PAMPS disintegration ratio (DR) was calculated as DR = ((*m*_0_ − *m*_*d*_)/*m*_0_) × 100% (g/g), where the *m*_0_ and *m*_*d*_ are the weight of hydrogel (g) before and after disintegration, respectively.

### 2.4. Preparation of Seed Coating Agent

The SA-loaded PAMPS hydrogel (F8) was prepared. SA powders (0.05 g (S1), 0.10 g (S2), and 0.15 g (S3)) were weighted and dissolved in 100 mL of ddH_2_O separately. Next, 1.5 g (P1) or 2.5 g (P2) F8 PAMPS powder was added into the above three SA solutions for full swelling and were taken out after 24 h. These SA-loaded hydrogels (P1S1, P1S2, P1S3, P2S1, P2S2, and P2S3) were dried at 60°C for 48 h and ground into powder for further use. Eleven seed pelleting agents were prepared in this study according to the formula given in Table 2.

### 2.5. Preparation of Tobacco Pelleted Seeds

Two grams of tobacco seeds was first coated with pure water and the above pelleting agents in a cyclic alternating pattern until the seed size reached 1.00~1.25 mm diameter (5~8 mL water per gram of naked tobacco seeds sprayed in total); then a second layer of pelleting agents and adhesive solution were supplied cyclically until the seed size was 1.60~1.80 mm diameter. All seeds were pelleted by a minitype coater “BY300A” (Shanghai, China) and were air-dried for 2 days at room temperature.

### 2.6. Seed Germination and Seedling Growth under Drought

Pelleted seeds were placed one by one in each well of the plastic plate, which filled with disinfected soil matrix. Three replications of 100 seeds each for each treatment were used. And drought stress was imposed by adjusting the soil moisture content to 40% of soil maximum water content. Then, seeds were incubated in a growth chamber (DGX-800E, Safe Experiment Instrument Factory, China) with 250 *μ*mol*·*m^−2^ *·*s^−1^ light intensity and an alternating cycle of 8 h light at 30°C and 16 h darkness at 20°C for 16 days [18]. Since the second day of experiment, the germinated seeds were recorded daily for 16 days (standard germination is when the root tip was approximately 1 mm). Germination index (GI) and vigor index (VI) were calculated according to Hu et al. [19] as follows: GI = ∑Gt/Tt, where Gt is the number of the newly germinating seeds in times of Tt; *VI*⁡ = GI × seedling  dry  weight. In addition, root length and seedling length were manually measured with a ruler, and seedling dry weight was determined after drying at 80°C for 24 h [20]. These measurements were made on thirty randomly selected normal tobacco seedlings for each replicate [21].

### 2.7. Statistical Analysis

Statistical analysis of all data was done by means of analysis of variance (ANOVA) using SAS version 8.0 software (SAS Institute, Cary, NC). Fisher's least significant difference (LSD) tests (*P* < 0.05) were adopted for multiple comparison. The percent data were transformed according to *y* = arcsin [sqr(*x*/100)] before ANOVA.

## 3. Results

### 3.1. Fourier Transform Infrared Spectroscopy Analysis of PAMPS

**Figure 1:**
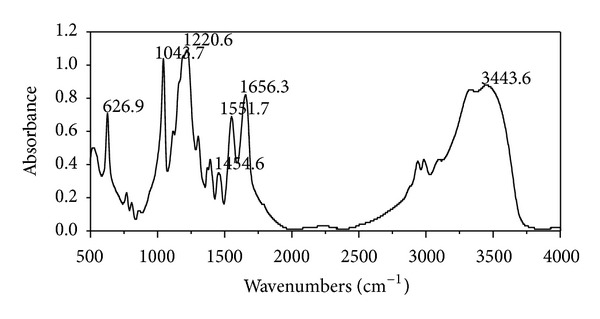
FTIR spectrum of PAMPS hydrogel.

A typical spectrum of PAMPS copolymer was shown in Figure 1. The characteristic absorption peak of AMPS units was shown at 1220.6 cm^−1^ due to the S=O group. The peak was shown at 1043.7 cm^−1^ due to the sulfonic acid groups. The amide I peak at 1656.3 cm^−1^ (C=O stretching) and amide II peak at 1551.7 cm^−1^ (N–H bending vibration) were ascribed to the amide bond of PAMPS. The N–H stretching peak was shown at 3443.6 cm^−1^.

### 3.2. The Liquid Absorption Performance

**Figure 2:**
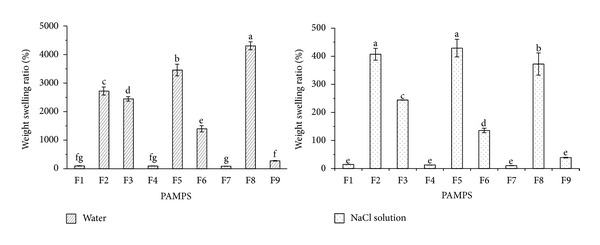
Weight swelling ratios of PAMPS hydrogels in water and salt solution for 24 h at 25°C. Lowercased letters mean significant difference (a = 0.05, LSD) among different PAMPS hydrogels. The formulas of different PAMPS hydrogels are shown in Table 1. Error bars represent ±S.E.

After 24 h liquid swelling, weight swelling ratios (SR) of nine PAMPS hydrogels were shown in Figure 2. Based on 35 g AMPS and 0.0105 g MBA, no matter how much KPS was applied, F1, F4, and F7 had lower weight swelling ratios in water and salt solution compared with other prepared PAMPS hydrogels; meanwhile, there were no significant differences among these three hydrogels. However, F2, F5, and F8, after increasing the amount of MBA to 0.0350 g, had higher SRs than other PAMPS hydrogels; and their SRs increased obviously with the increasing of KPS amount when water is used as a swelling medium. In addition, it was worth noting that the SRs of PAMPS hydrogels (F3, F6, and F9) turned to decline if too much MBA (0.105 g) were added into the polymerized system, and their SRs decreased quickly as the KPS amount increased.

**Figure 3:**
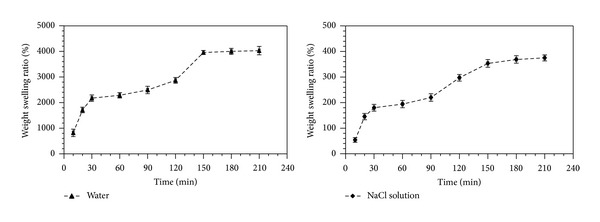
Weight swelling kinetics of F8 PAMPS polymers in water and salt solution at 25°C. F8 (shown in Table 1) is the PAMPS hydrogel synthesized by 35 g of AMPS (2-acrylamido-2-methylpropane sulfonic acid), 0.021 g of KPS (potassium persulfate), and 0.035 g of MBA (N, N′-methylene-bis(acrylamide)). Error bars represent ±S.E.

The PAMPS polymerized according to the F8 formula in Table 1 had the highest SR in water and reached 4306 times its own dry weight. Its SR in salt solution was 373, which was a little lower than the SR of F2 and F5 PAMPS. Thus, the F8 PAMPS was chosen to determine the change of weight swelling ratio along with the increasing of liquid absorption time at 25°C (Figure 3). The SR of F8 PAMPS in water and salt solution increased obviously and reached more than 4000 and 350 times its own dry weight, respectively, after 3 h. In addition, the weight swelling rates during the first 30 min of liquid absorption were evidently faster than other time periods.

### 3.3. The Water Retention Properties

**Figure 4:**
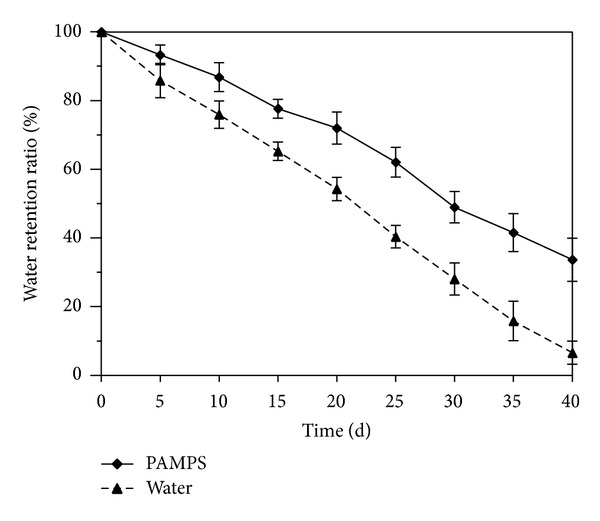
Water retention kinetics of F8 PAMPS polymer at 25°C. Error bars represent ±S.E. For additional explanations, see Figure 3.

**Figure 5:**
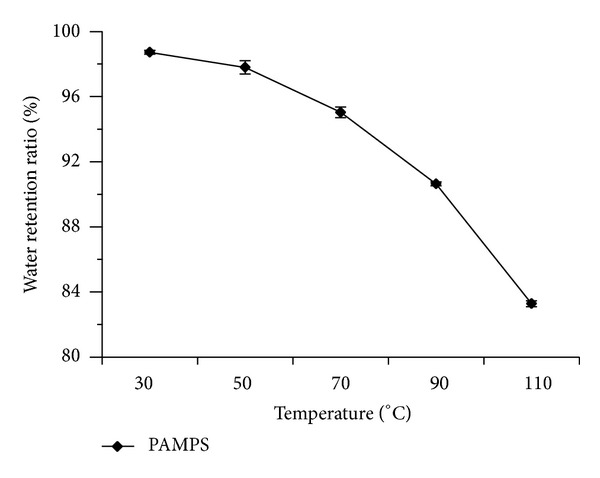
Water retention ratios of F8 PAMPS polymer at different temperature for 30 min. Error bars represent ±S.E. For additional explanations, see Figure 3.

The water retention properties of PAMPS hydrogel (F8) were tested under room temperature (25°C) (Figure 4) and higher temperatures (Figure 5). The results showed that the water retention ratio (RR) of PAMPS (F8) at a given time was always higher than that of control at 25°C. Compared with the 6.6% of original weight of control after 40 d, the F8 PAMPS hydrogel could keep the RR as high as 33.67% (Figure 4). Even treated under 110°C for 30 min, the PAMPS still kept RR at 85.3% (Figure 5).

### 3.4. The Disintegration Characteristic

**Figure 6:**
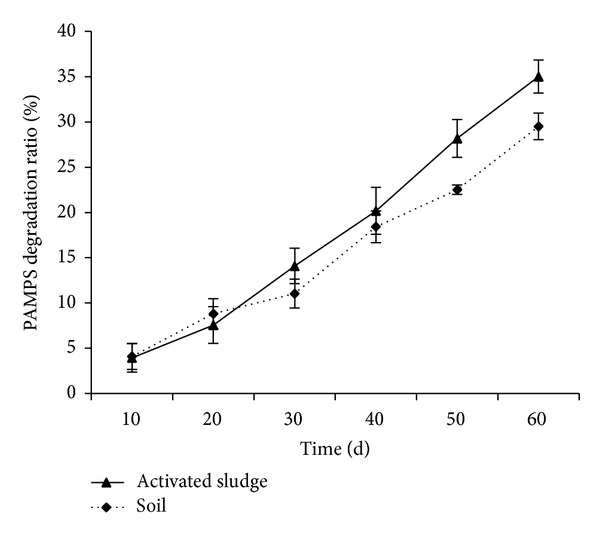
F8 PAMPS polymer disintegration ratio versus time histories in soil and activated sludge. Error bars represent ±S.E. For additional explanations, see Figure 3.

The F8 PAMPS disintegration ratio (DR) increased gradually as the time went on (Figure 6). After embedding in soil and activating sludge for 60 d, the PAMPS disintegration ratio was 29.5% and 35%, respectively. Moreover, except for 20 d, The F8 PAMPS hydrogel had higher DR in activated sludge than in soil.

### 3.5. Changes of Seed Germination and Seedling Growth under Drought Condition

For both of tobacco varieties, except S1 and S3, other nine treatments significantly improved the seed germination under drought stress compared with the control (Table 3). All of the GP, GI, and VI of P2S3 had the highest value in the above nine pelleting treatments, especially for HHDJY. For MSk326, except the GP of P2S3 which was a little lower than that of P2S2, the GI and VI were still higher than other treatments.

No significant differences were found in root length (RL), seedling length (SL), and dry weight (SDW) among the treatments with P1, P2, S1, and the control in both of tobacco varieties (Tables 4 and 5). However, three treatments with 2.5 g of PAMPS loaded with SA (P2S1, P2S2, and P2S3) in HHDJY and four treatments (P1S3, P2S1, P2S2, and P2S3) in MSk326 significantly improved the RL, SL, SFW, and SDW as compared with the control, and the most pronounced effect was recorded in P2S3. In addition, seed pelleted with S3 inhibited significantly the seedling growth compared with the control and other treatments irrespective of variety.

## 4. Discussions

In this study, the best super absorbent PAMPS hydrogel according to 1 : 0.00046 : 0.00134 (mol/mol/mol) ratios of the AMPS monomer to KPS and MBA was prepared. It is worth noting that when appropriate MBA was applied, the liquid absorption ratio of PAMPS could be improved by increasing KPS; however, it would be inhibited after improper amount of BMA was used. The results suggested that MBA played an important role in the preparation of PAMPS hydrogels having less or more density network, which is responsible for the swelling degree of the obtained hydrogels. Also, Nalampang et al. [22] found the significant influence of crosslink density (MBA) on water content of AMPS-based hydrogels.

The major FTIR absorption peaks of the PAMPS were consistent with related reports [17] and there was no other impurity peaks. It was reasonable to conclude that the PAMPS was polymerized successfully according to the synthesis route. The water absorption ratio could reach as much as 4306 times its own dry weight, which was far higher than other materials such as keratin-based hydrogel [3], polyacrylic acid sodium salt [4], and starch-based superabsorbent [6]. It might be because the linear polymers with sulfonate groups derived from AMPS exhibit extensive coil expansion in aqueous solutions, even in a 5 M NaCl solution [17]. In addition, due to the strongly ionizable sulfonate group, AMPS dissociates completely in the overall pH range, and the hydrogels derived from AMPS exhibit pH independent swelling behavior [23]. Thus, the prepared PAMPS hydrogel in this study was supposed to be able to adapt to various pH conditions. However, it still warrants further study.

PAMPS exhibited slow disintegration characteristic in soil; two months later, about one-third of PAMPS weight degraded and eventually broke down for nitrogen dioxide, water, ammonia nitrogen, and sodium ion, and so forth. That meant it was nontoxic and environmentally safe to be used [24]. Meanwhile, the above characteristic of PAMPS enhanced its valid period and efficiency in practice. Up to now, productions of super absorbent polymers in China have been widely used in food crops, economic crops, vegetables, flowers, fruit trees, lawn cultivation, and so forth and proved to be effective [24].

However, it was still unclear whether the PAMPS was suitable for pelleting or could enhance tobacco seed drought resistance as a small “reservoir” without interference of coating agents. Therefore, studying the effects of different coating agents including PAMPS on tobacco seedling establishment was necessary. The results showed that the PAMPS alone (P1 and P2) could improve the seed germination and seedling growth under drought, even the increase of seed GP, GI, and VI reached a significant level as compared with the control. It suggested that the moisture absorbed by polymer from soil could be used gradually at stages of seedling growth. The similar results were also reported in soybean seed coated with starch-grafting super absorbent resin [2] and maize seed coated with polyacrylic acid sodium high water absorbent polymer [25].

Moreover, the P2S3, 2.5 g PAMPS loaded with 0.15 g SA, was proved to be the best coating agent formula for tobacco seed pelleting. The positive effect of SA-loaded PAMPS on seed germination and seedling growth under drought stress was better than that of the SA or PAMPS alone. Salicylic acid improves germination by neutralizing free radicles or active oxygen; however, it was important to note that 0.15 g SA alone had an obvious inhibition on seedling growth; however, after loading the same amount of SA into PAMPS, P1S3 and P2S3 distinctly turned to improve the seed germination. This might be due to the high concentration of SA that negatively influences the plant growth via inhibiting the activities of protective enzymes and promoting MDA production [26]. Meanwhile, the PAMPS hydrogel might have the controlled effect on SA release to some extent to ensure the lower effective concentration instead of the higher inhibiting concentration.

In addition, it suggested that the likely reasons for drought tolerance obtained by tobacco pelleting seeds with PAMPS coating agents were as follows: one was that more than 85% water absorbed by super absorbent polymer was considered to be effective to the plant. Like a miniature reservoir, as the rhizosphere soil moisture changed, PAMPS could absorb and release water repeatedly to supply for plant roots utilization [27]. Another study showed that the soil structure was improved via PAMPS application, which contributed to the improvement of soil porosity and air permeability [28, 29]. Su et al. [30] reported that as a result of water in the soil absorbed by hydrogel, soil thermal conductivity, and temperature difference between day and night reduced, which would be good for the crop growth.

In this study, PAMPS was used not only as superabsorbent polymer but also as SA controlled release material that was given full play to the dual-use value of PAMPS. On the one hand, water released from PAMPS improved tobacco seedling establishment under drought; on the other hand, the SA loaded in the PAMPS would be controlled released to further enhance the seedling drought resistance.

## 5. Conclusions

In practice, with the special “reservoir” characteristic, the SA-loaded PAMPS hydrogel represents a potential dual-effect on improvement of tobacco seed and seedling drought tolerance. This seed pelleting method with enhanced drought-resistant function may become a novel pathway to enhance seed establishment under stresses. However, this method still should be verified in other crop seeds; or there should be an upgrade to this method with different chemicals to implement in plant resistance against stresses.

## Figures and Tables

**Table 1:** Preparation of poly(2-acrylamido-2-methylpropane sulfonic acid) (PAMPS) hydrogels.

Sample code	AMPS∗ (g)	KPS (g)	MBA (g)
F1	35	0.007	0.0105
F2	35	0.007	0.0350
F3	35	0.007	0.1050
F4	35	0.014	0.0105
F5	35	0.014	0.0350
F6	35	0.014	0.1050
F7	35	0.021	0.0105
F8	35	0.021	0.0350
F9	35	0.021	0.1050

∗AMPS is 2-acrylamido-2-methylpropane sulfonic acid; KPS is potassium persulfate; MBA is N, N′-methylene-bis(acrylamide).

**Table 2:** The different formulas of tobacco seed pelleting agents.

Treatment∗	PAMPS (g)	SA (g)	Talc (g)	Bentonite (g)	Total weight (g)
Control	0	0	70.00	30	100
P1	1.5	0	68.50	30	100
P2	2.5	0	67.50	30	100
S1	0	0.05	69.95	30	100
S2	0	0.10	69.90	30	100
S3	0	0.15	69.85	30	100
P1S1	1.5	0.05	68.95	30	100
P1S2	1.5	0.10	68.40	30	100
P1S3	1.5	0.15	68.35	30	100
P2S1	2.5	0.05	67.45	30	100
P2S2	2.5	0.10	67.40	30	100
P2S3	2.5	0.15	67.35	30	100

∗PAMPS is poly(2-acrylamido-2-methylpropane sulfonic acid) hydrogel (F8) and SA is salicylic acid, tobacco seed pelleting agents with different formulas; the control is the mixture of talc and bentonite without PAMPS hydrogel and SA; P1 and P2 are mixtures of PAMPS hydrogel, talc and bentonite without SA; S1 and S2 are mixtures of SA, talc, and bentonite without PAMPS hydrogel; P1S1, P1S2, P1S3, P2S1, P2S2, and P2S3 are mixtures of SA-loaded PAMPS hydrogel, talc, and bentonite. F8 (shown in Table 1) is the PAMPS hydrogel synthesized by 35 g of AMPS (2-acrylamido-2-methylpropane sulfonic acid), 0.021 g of KPS (Potassium persulfate), and 0.035 g of MBA (N, N′-methylene-bis (acrylamide)).

**Table 3:** Effects of different coating agents on tobacco pelleting seed germination under drought stress.

Treatment^b^	HHDJY			MSk326		
	GP (%)	GI	VI	GP (%)	GI	VI
Control	54.2 c^a^	2.84 c	47.21 c	52.3 c	2.73 c	43.30 d
P1	63.4 b	3.38 b	56.77 b	62.5 ab	3.32 b	54.02 c
P2	66.7 b	3.55 b	59.74 b	63.4 ab	3.41 b	55.67 c
S1	56.7 c	2.92 c	50.68 c	50.7 c	2.81 c	40.05 d
S2	64.7 b	3.45 b	58.21 b	63.3 b	3.41 b	57.26 bc
S3	40.7 d	2.13 d	38.86 d	43.3 e	2.25 d	40.79 e
P1S1	70.8 ab	3.79 ab	65.35 b	65.3 ab	3.48 ab	56.81 c
P1S2	70.8 ab	3.89 ab	66.61 ab	65.7 ab	3.55 ab	57.96 bc
P1S3	67.6 b	3.66 b	62.96 b	65.3 ab	3.43 b	57.38 bc
P2S1	71.8 ab	3.85 ab	66.76 ab	63.9 b	3.43 b	57.16 bc
P2S2	72.2 ab	3.93 ab	68.08 ab	68.1 a	3.68 ab	61.66 ab
P2S3	73.6 a	4.07 a	71.11 a	67.1 ab	3.70 a	63.32 a

GP: germination percentage; GI: germination index; VI: vigor index.

^a^Lowercased letters mean significant difference (a = 0.05, LSD) among treatments within the same variety.

^b^For additional explanations, see Table 2.

**Table 4:** Effects of coating agents on seedling growth of Honghuadajinyuan (HHDJY) under drought stress.

Treatment^b^	RL (mm)	SL (mm)	SFW (mg/30 plants)	SDW (mg/30 plants)
Control	8.02 c^a^	15.75 b	141.5 b	15.84 c
P1	8.66 bc	16.80 ab	147.8 b	16.68 bc
P2	8.87 bc	16.82 ab	150.4 ab	16.86 bc
S1	8.13 c	15.66 b	146.5 b	15.57 c
S2	9.15 ab	16.80 ab	154.3 ab	17.40 ab
S3	7.56 d	14.38 c	130.9 c	14.85 d
P1S1	9.10 ab	17.22 a	157.5 a	17.49 ab
P1S2	8.93 bc	17.12 a	154.5 ab	16.65 bc
P1S3	9.16 ab	17.19 a	153.0 ab	17.34 ab
P2S1	9.13 ab	17.34 a	157.5 a	17.49 ab
P2S2	9.17 ab	17.34 a	158.3 a	17.64 ab
P2S3	9.38 a	17.46 a	161.3 a	17.85 a

RL: root length; SL: seedling length; SFW: seedling fresh weight; SDW: seedling dry wight.

^a^Lowercased letters mean significant difference (a = 0.05, LSD) among treatments within the same variety.

^b^For additional explanations, see Table 2.

**Table 5:** Effects of coating agents on seedling growth of MSk326 under drought stress.

Treatment^b^	RL (mm)	SL (mm)	SFW (mg/30 plants)	SDW (mg/30 plants)
Control	7.55 c^a^	14.38 c	130.9 c	15.09 c
P1	8.39 bc	16.28 bc	139.7 b	16.14 c
P2	8.42 bc	16.34 bc	142.0 b	16.26 c
S1	7.50 c	14.45 c	132.7 c	16.05 c
S2	8.41 bc	16.31 bc	141.8 b	16.74 b
S3	7.02 d	13.56 d	119.6 d	14.19 d
P1S1	8.48 bc	16.31 bc	149.3 b	15.99 c
P1S2	8.49 bc	16.32 bc	151.5 ab	16.80 b
P1S3	8.78 b	16.73 ab	150.8 ab	17.10 ab
P2S1	8.85 ab	16.65 ab	150.8 ab	17.04 ab
P2S2	8.83 ab	16.78 ab	152.3 ab	17.40 a
P2S3	9.16 a	17.13 a	158.3 a	17.40 a

RL: root length; SL: seedling length; SFW: seedling fresh weight; SDW: seedling dry weight.

^a^Lowercased letters mean significant difference (a = 0.05, LSD) among treatments within the same variety.

^b^For additional explanations, see Table 2.
